# Farmland change and its implications in the Three River Region of Tibet during recent 20 years

**DOI:** 10.1371/journal.pone.0265939

**Published:** 2022-04-11

**Authors:** Hui Wei, Changhe Lu

**Affiliations:** 1 Key Laboratory of Land Surface Pattern and Simulation, Institute of Geographic Sciences and Natural Resources Research, CAS, Beijing, China; 2 University of Chinese Academy of Sciences, Beijing, China; Northeastern University (Shenyang China), CHINA

## Abstract

Farmland is a key resource for safeguarding the regional food security and social stability, particularly in Tibet where the farmland is very limited due to its high altitude. With quick economic development during recent decades, farmland changes are great in China, and thus have been extensively studied. These studies generally focused on eastern regions, and seldom for Tibet due to the lack of good quality and available data. To this end, taking the Three River Region (TRR) as the case area, this study obtained 1 m spatial resolution farmland data for 2000 and 2018 by visual interpretation of the Google Earth high resolution satellite images, and then analyzed the farmland change, its driving factors and impact on grain production between 2000 and 2018. The results showed that farmland in the TRR decreased by 8.85% from 219.29 k ha in 2000 to 199.89 k ha in 2018, averagely reduced by 0.51% per year, mainly driven by the economic development, agricultural progress, urbanization, and population growth. The farmland losses largely occurred in urban areas and their surrounding counties due to urban land occupation, and caused the grain production reduced by 9.38%. To control the quick farmland losses and to ensure the regional food security of Tibet, it should strengthen the supervision on non-agricultural occupation of farmland and increase agricultural investment to improve the land productivity in the TRR.

## Introduction

Farmland is a significant support for ensuring regional food security and maintaining regional economic and social stability [1, 2]. According to the data released by UNESCO and FAO, global total farmland area increased from 1.28 billion ha in 1961 to 1.73 billion ha in 2015, while per capital farmland area dropped from 0.42 ha to 0.26 ha due to rapid population growth [3]. Recently published farmland area in China based on the third national land survey was totaled 128 million ha, averaged 0.09 ha per capita, only 34.62% of the world average. Due to non-agricultural occupation induced by rapid economic development and urbanization, farmland in China has experienced a quick decrease in the quantity [4, 5] and quality [6] during recent decades. These have raised great concerns on its protection and sustainable utilization [7–9], and thus promoted a large number of relevant researches. The studies addressed the spatiotemporal change [10–16], driving factors [9, 12, 13, 17], and its impact on grain production [1, 5, 10], mainly in the areas of rapid economic development, such as the Yangtze River Delta [10], the Pearl River Delta [11], and the Beijing-Hebei-Tianjin region [12], and the main grain producing areas of Henan [13], Hebei [14], and Shandong provinces [15]. The data used in the studies were mainly collected from yearbooks [16, 17] or land use remote sensing data products [5, 10–15] at a coarse spatial resolution ranging from 10 m to 8 km [18], and thus often involved uncertainties, particularly for the areas of fragmented landscape.

Tibet Autonomous Region, located in the south Tibetan Plateau, is one of important pasturing regions in China. Restricted by fragile environment of high altitude and fragmented landscape, the farmland resource is limited, covering only 0.18% of the total land area and mainly distributing in valley areas. During recent decades, associated with the rapid urbanization and population growth [19], Tibet showed a significant land use change and farmland loss [20], threatening the food security and ecological safety [21, 22]. To date, only four studies addressed specifically the farmland changes in Tibet, based on the farmland data either collected from the local yearbooks [17] or Landsat image derived maps [23–25]. Due to limitation of data used in these studies, the amplitude and spatial variation of farmland changes in Tibet were generally not accurately captured, and thus a further analysis is needed based on more reliable, such as high spatial resolution farmland data. Presently, the 30 m land use data interpreted from the Landsat images, as released by the Resource and Environment Science and Data Center of the Chinese Academy of Sciences [26], are the highest resolution long-term series data products available for whole China. Another series data product is the detailed nationwide land use survey data, accomplished by the Chinese government in 1997, 2009 and 2019, respectively, using high-resolution images in combination with air photos and field surveys, but the spatial data have not been released to the public. Therefore, we collected available 0.51–1.02 m resolution images from Google Earth for the case area of the Three River Region (TRR), the core grain production area of Tibet, and extracted the accurate farmland data for 2000 and 2018. With these interpreted data, we aimed to identify the amplitude and driving factors of farmland change during 2000 to 2018, as well as its impact on grain production, using a combination method of GIS, principal component analysis, and multiple linear regression analysis. This study produced the firsthand farmland data of 1 m spatial resolution and filled the data gap, and thus provides a support for the farmland protection and sustainable grain production in the TRR.

## Materials and methods

### Study area

**Fig 1:**
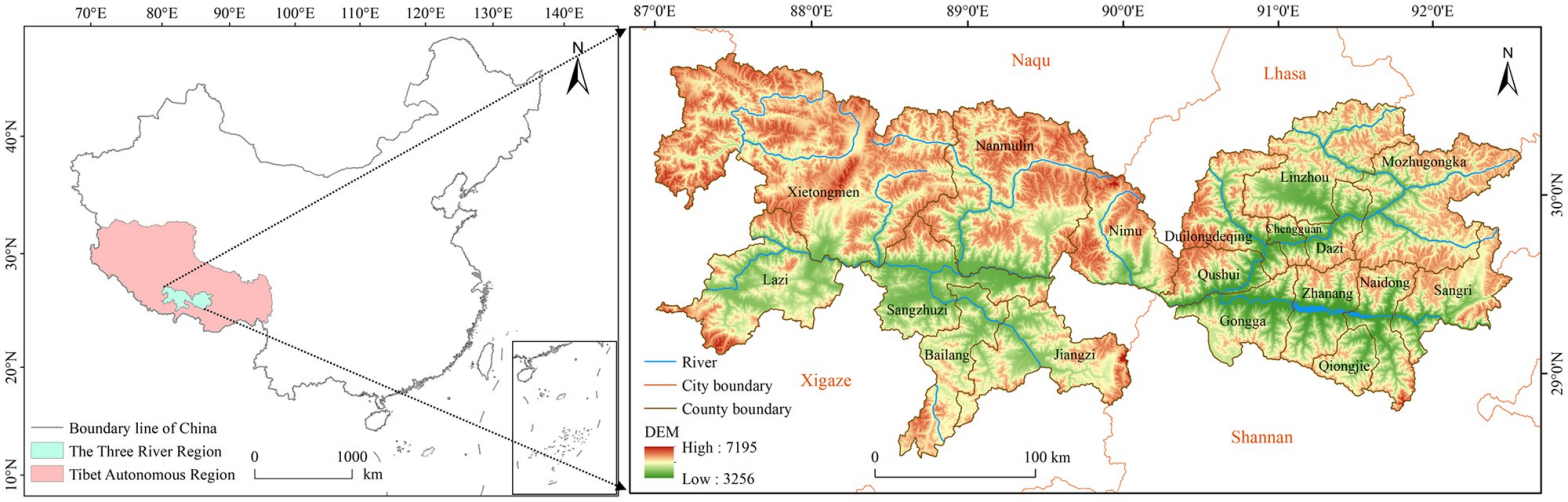
Location and terrain map of the Three River Region. The map was prepared with ArcGIS10.1 (https://resources.arcgis.com/en/help/), based on the maps of China political boundaries and rivers from the Resource and Environment Science and Data Center, CAS (https://www.resdc.cn/), with the permission of its copyright, and ASTER Global Digital Elevation Model V2 [28] courtesy of NASA Earth Data (https://earthdata.nasa.gov/).

The TRR (28°20’– 31°20’ N, 87°00’– 92°35’ E) is located in the middle reaches of the Yarlung Zangbo River, covering valley areas of the river and its tributaries, including the two major ones, the Lhasa River and the Nianchu River. The TRR has an area of 67,949 km^2^, involving three prefecture-level cities of Lhasa, Xigaze and Shannan, and 18 county-level administrative units. The region is acknowledged as the granary of Tibet [27], occupying 46.84% of the farmland area and producing 52.32% of the total grain output in 2018. The terrain comprises river valleys or basins with the altitude mostly at 2700–4600 m a.s.l. in the middle, and high mountains in the north and south (Fig 1). It has a semi-arid temperate monsoon climate, characterized by warm summer, and clod and dry winter [27]. The mean temperature for annual, July, and January in the valley areas is 4–9°C, 10–16°C, and -12–0°C, respectively. Influenced by the high altitude, the TRR has strong solar radiation, with the annual sunshine hours ranging 2800–3300 h, and radiation intensity ranging 6670–8074 MJ/m^2^. As a result, the diurnal variation of temperature is high, normally above 14°C. The annual mean precipitation is 250–580 mm, with 77–93% falling in the rainy season of June to September. Main growing crops are naked highland barley, spring wheat, oilseed rape, potatoes, normally growing in the valley areas below the altitude of 4600 m a.s.l.

### Data sources

The data used in this study include: 1) vector map of the TRR, extracted from the China administrative map of counties; 2) 0.51–1.02 m resolution satellite images, derived from the open-access Google Earth database; 3) daily meteorological data at 20 stations in and around the TRR from 2000 to 2018, obtained from the Resource and Environment Sciences and Data Center of the Chinese Academy of Sciences [29]; 4) 30 m resolution DEM data of the open-access ASTER GDEM Version 2 [28]; 5) county-level socioeconomic statistical data from 2000 to 2018, collected from Yearbooks of Tibet Autonomous Region, County Yearbook of China, and Statistical Bulletin of National Economic and Social Development at County Level.

### Research method

#### Data acquisition and spatiotemporal analysis of farmland

The farmland in 2000 and 2018 were obtained by visual interpretation of the high-resolution images. Based on available imagery data, 77.48% of the farmland in 2018 was interpreted from the images of July 2018 to December 2018; 16.55% and 3.76% from the images of January 2017 to December 2017, and January 2019 to June 2019, respectively; and the remaining 2.21% was obtained from the images of May 2010 to December 2013, due to the lack of recent images. In 2000, 63.88% of the farmland was interpreted from images of May 2000 to December 2000; 28.35% from images of November 2001 to December 2002; and the remaining 7.78% was obtained from the images of November 2003 to December 2007.

The interpreted data were saved as kml files, and then imported to ArcGIS10.6 to calculate the farmland area in 2000 and 2018, respectively. By overlaying the two farmland maps with ArcGIS, the change of farmland area between 2000 and 2018 in the TRR was obtained. Further, we applied the compound interest formula in economics [30] to calculate the annual farmland change rate during 2000 to 2018, as below:

$$k=\left[{\left(\frac{U_{b}}{U_{a}}\right)}^{\frac{1}{T}}-1\right]\times 100\%$$ (1)

where, *k* is the annual farmland change rate during 2000–2018; *U*_*a*_ and *U*_*b*_ are the farmland area in 2000 and 2018, respectively; and *T* is the total years of the study period.

#### Identification of driving factors of farmland change

In this study, 9 factors were determined to identify the contribution to farmland change in the TRR, comprising thSe main social-economic factors, of which the data are complete during 2000–2018. These factors included gross domestic product (GDP) (X1), total investment in fixed assets (X2), local fiscal expenditure (X3), rural disposable income per capita (X4), urbanization rate (X5), total population (X6), grain crop yield (X7), total agricultural machinery power (X8), and agricultural output value ratio to GDP (X9).

The principal component analysis (PCA) was chosen to identify the driving factors of farmland change. The PCA is a tool to recombine the correlated factors into several independent principal components (PC), each of which is a linear combination of the original factors and has a significant joint influence [31]. The PC was determined based on the eigenvalue (EV) and principal component contribution rate (PCR), as calculated with SPSS 19.0. According to the Kaiser principle [32], for the EV exceeding 1 and total PCR exceeding 85%, each of the combinations of original factors is identified as a PC, indicating that the involved factors have a significant influence on farmland change. The mathematical model of PCA is as follows [31]:

$$F_{i}=a_{i1}ZX_{1}+a_{i2}ZX_{2}+\cdots a_{ij}ZX_{j}$$ (2)

$$a_{ij}=\frac{L_{ij}}{\sqrt{EV_{i}}}$$ (3)

$$F=c_{1}F_{1}+c_{2}F_{2}+\cdots c_{n}F_{n}$$ (4)

$$c_{i}=\frac{PCR_{i}}{\sum_{i=1}^{n}PCR_{i}}$$ (5)

$$CR_{i}=\frac{c_{i}}{\sum_{i=1}^{j}\left|c_{i}\right|}$$ (6)

where, *F*_*i*_ and *F* are the score for the *i*-th principal component and the comprehensive score for driving factors of farmland change, respectively; *ZX*_*j*_ is the normalized value of the *j*-th factor; *a*_*ij*_ is the correlation coefficient between the *j*-th factor and the *i*-th principal component, *i* ≤ *j*; *L*_*ij*_ is the load value of the *i*-th principal component on the *j*-th factor; *EV*_*i*_ and *PCR*_*i*_ are the eigenvalue and contribution rate of the *i*-th principal component, respectively; *n* is the number of principal component; and *CR*_*i*_ is the contribution rate of the *i*-th factor.

#### Impact of farmland change on grain production

Total grain production is a product of the mean grain crop yield and total sown area [1]. As crop sown area is highly related to the farmland area, impact of farmland change on grain production can be estimated by regression analysis. Therefore, we selected farmland area change and mean grain yield change for each county as independent variables, and total grain production change as the dependent variable, and quantified their relationship by multivariate linear regression (MLR) [33]. Firstly, we calculated the change ratios (as % of that in 2000) in the total grain production, mean grain yield and farmland area for each of the 18 counties between 2000 and 2018, and then conducted the MLR analysis using the SPSS19.0. The general formula [33] is as follow:

$$\Delta G_{i}=a\Delta Y_{i}+b\Delta F_{i}+c$$ (7)

where, Δ*G*_*i*_ is the change (%) of total grain production; Δ*Y*_*i*_ and Δ*F*_*i*_ are the change of mean grain yield and farmland area in the *i*-th county, respectively; *a* and *b* are the regression coefficients, respectively; and *c* is the constant term.

## Results

### Farmland change and its spatial variation

The total farmland area in the TRR was 219.29 k ha in 2000 and 199.89 k ha in 2018, reduced by 19.40 k ha or 8.85% between 2000 and 2018, averaged -0.51% per year (Table 1). In 6 counties/districts of Chengguan, Duilongdeqing, Sangri, Linzhou, Sangzhuzi, and Jiangzi, the farmland area was significantly reduced by 16.88–52.22%, averaged more than 1.0% per year. In 6 counties of Qiongjie, Nimu, Naidong, Mozhugongka, Lazi, and Xietongmen, it was reduced by 0.7–15.19% or 0.04–0.91% per year. In the remaining 6 counties, it was expanded by 1.19–11.16% or 0.07–0.59% per year.

**Table 1:** Change of farmland area in counties of the Three River Region from 2000 to 2018.

County	Farmland area (k ha)		Area change (%)	Annual change rate (%)
	2000	2018		
Chengguan	2.79	1.34	-52.22	-4.02
Duilongdeqing	10.00	6.56	-34.36	-2.31
Sangri	4.50	3.33	-25.86	-1.65
Linzhou	21.58	17.29	-19.87	-1.22
Sangzhuzi	33.46	27.64	-17.41	-1.06
Jiangzi	22.87	19.01	-16.88	-1.02
Qiongjie	4.17	3.54	-15.19	-0.91
Nimu	5.03	4.41	-12.36	-0.73
Naidong	9.07	8.56	-5.64	-0.32
Mozhugongka	13.42	12.93	-3.65	-0.21
Lazi	17.91	17.44	-2.66	-0.15
Xietongmen	7.58	7.52	-0.70	-0.04
Dazi	8.38	8.48	1.19	0.07
Gongga	11.09	11.35	2.35	0.13
Zhanang	7.96	8.29	4.12	0.22
Bailang	14.97	15.65	4.54	0.25
Nanmulin	16.68	17.85	7.04	0.38
Qushui	7.83	8.71	11.16	0.59
Total	219.29	199.89	-8.85	-0.51

**Fig 2:**
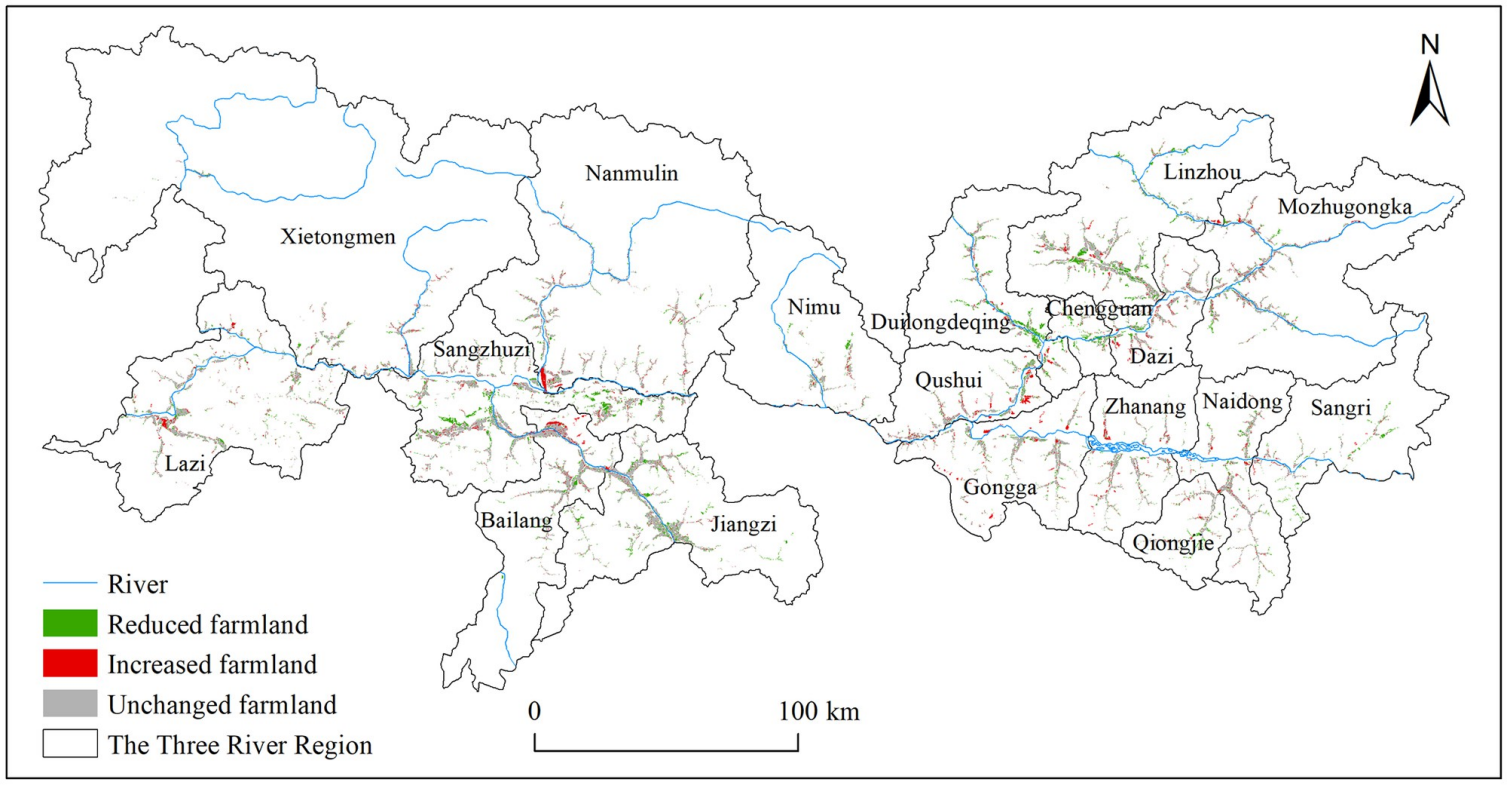
Farmland changes between 2000 and 2018 in the Three River Region. The map was prepared with ArcGIS10.1 (https://resources.arcgis.com/en/help/), based on the maps of China political boundaries and rivers from the Resource and Environment Science and Data Center, CAS (https://www.resdc.cn/), with the permission of its copyright, and farmland data from Wei et al., [34] (https://doi.pangaea.de/10.1594/PANGAEA.937400).

From 2000 to 2018, 77.12% of the total farmland in 2000 was not changed and 22.88% (50.17 k ha) was converted to built-up land or other land use types. In addition, 30.77 k ha was newly reclaimed from grassland (Fig 2). The reduced farmland largely occurred in the main urban areas of the three prefecture-level cities and their surrounding counties, such as Chengguan, Duilongdeqing, Linzhou, and Gongga of Lhasa City; Sangzhuzi, Jiangzi, and Bailang of Xigaze City; and Naidong and Sangri of Shannan City; the reduced farmland area in these counties accounted for 84.24% of the total reduced farmland area in the TRR. The newly reclaimed farmland was mainly distributed in rural areas of main grain producing counties, such as Nanmulin, Bailang, Lazi, Qushui, Gongga, and Linzhou, accounting for half of the total newly reclaimed farmland area.

### Driving forces of farmland changes

As shown in Table 2, the absolute value of correlation coefficient exceeds 0.5 for most pair factors, indicating a significant repetitiveness of information expressed by original factors. The KMO (Kaiser-Meyer-Olkin) value was 0.74, larger than 0.50, and the Sig. value of Bartlett test was 0.00, less than the significance level of 0.05, implying that the PCA is suitable for identifying the principal components [31], i.e., the main driving forces of farmland change in the TRR.

**Table 2:** Correlation coefficient matrix among the selected factors.

**Factors**	**ZX1**	**ZX2**	**ZX3**	**ZX4**	**ZX5**	**ZX6**	**ZX7**	**ZX8**	**ZX9**
ZX1	1.000								
ZX2	0.979	1.000							
ZX3	0.987	0.994	1.000						
ZX4	0.978	0.948	0.967	1.000					
ZX5	0.773	0.685	0.709	0.816	1.000				
ZX6	0.509	0.464	0.462	0.518	0.812	1.000			
ZX7	0.221	0.367	0.338	0.205	-0.273	-0.380	1.000		
ZX8	0.910	0.851	0.888	0.968	0.856	0.540	0.116	1.000	
ZX9	-0.743	-0.633	-0.664	-0.781	-0.939	-0.695	0.278	-0.819	1.000

Two independent principal components were obtained, and their eigenvalue is 6.54 and 1.78, and the contribution rate is 72.65% and 19.81%, respectively. In sum, both principal components together explained 92.46% of the cause of farmland change during 2000–2018 in the TRR. For the first principal component (PC1), the absolute load value of X4, X1, X8, X3, X2, and X5 all exceeds 0.90, and that of X9 and X6 exceeds 0.80 and 0.60, respectively (Table 3), implying the PC1 well represents the combined effect of economic development (X4, X1, X3, X2), agricultural progress (X8), urbanization (X5), and population growth (X6). The second principal component (PC2) was strongly related to X7, with the load value of 0.94, indicating that the PC2 mainly represents the effect of agricultural progress, i.e., grain yield level (X7). In conclusion, the farmland change from 2000 to 2018 in the TRR was comprehensively affected by the economic development, agricultural progress, urbanization, and population growth.

**Table 3:** Load matrix of the principal components.

Factors	Principal components	
	PC1	PC2
Gross domestic product (X1)	**0.97**	0.20
Total investment in fixed assets (X2)	**0.92**	0.33
Local fiscal expenditure (X3)	**0.94**	0.31
Rural disposable income per capita (X4)	**0.98**	0.16
Urbanization rate (X5)	**0.90**	-0.40
Total population (X6)	0.66	-0.57
Grain crop yield (X7)	0.08	**0.94**
Total agricultural machinery power (X8)	**0.96**	0.05
Agricultural output value ratio to GDP (X9)	**-0.86**	0.39

To further compare the contribution of different factors, the linear relationship equation between the two PCs and the normalized value of original 9 factors were obtained, based on the SPSS analysis results. On this basis, a comprehensive score equation including all factors affecting farmland change was obtained, as shown in Eq (8), according to the contribution rate of two principal components to farmland change.


$$\begin{array}{ll} F & =0.79F_{1}+0.21F_{2}\\ & =0.33ZX_{1}+0.34ZX_{2}+0.34ZX_{3}+0.33ZX_{4}+0.21ZX_{5}+0.11ZX_{6}+0.18ZX_{7}+0.30ZX_{8}-0.20ZX_{9} \end{array}$$ (8)

The independent variables in Eq (8) are normalized values of the original factors, and thus the coefficient can represent the relative contribution of corresponding factor. Based on Eq (6), we calculated contribution rate of each factor, and found that local fiscal expenditure (X3), total investment in fixed assets (X2), GDP (X1), rural disposable income per capita (X4), and total agricultural machinery power (X8) were the top five contributing factors, and their contribution rate was 14.49%, 14.43%, 14.06%, 13.98%, and 12.89%, respectively. Urbanization rate (X5), grain crop yield (X7) and total population (X6) also had a positive contribution to the farmland change, but the contribution rate was lower, at 9.13%, 7.51%, and 4.85%, respectively. Agricultural output value ratio to GDP (X9) played a negative role, contributing -8.65% to the farmland change.

### Impact of farmland change on grain production

The multivariate regression results indicate that between 2000 and 2018, the change in total grain production (Δ*G*) was significantly related to changes of mean grain yield (Δ*Y*) and farmland area (Δ*F*), with the coefficient of determination (*R*^2^) reaching 0.96 (Sig = 0). The fitting binary regression equation is as below:

$$\Delta G_{i}=0.84\Delta Y_{i}+1.06\Delta F_{i}-0.13$$ (9)

From this equation, it can be inferred that the total grain production would increase (decrease) 0.84% for each 1% increase (decrease) of mean grain yield when the farmland area remains unchanged, while it would increase (decrease) by 1.06% for 1% increase (decrease) of farmland area when the mean grain yield remains unchanged. From 2000 to 2018, the mean grain yield increased by 0.74–52.56% in 12 counties, and decreased by 2.15–58.0% in 6 counties, with the regional average increased by 11.79%, and the farmland area changed by -52.22–11.16% (Table 1), reduced by 8.85% for the whole TRR. By estimation based on Eq (9), the farmland change caused a total grain reduction by 9.38% or 50.35 k tons in the TRR.

## Discussion

### Interpreted data verification and comparison with existing data

**Fig 3:**
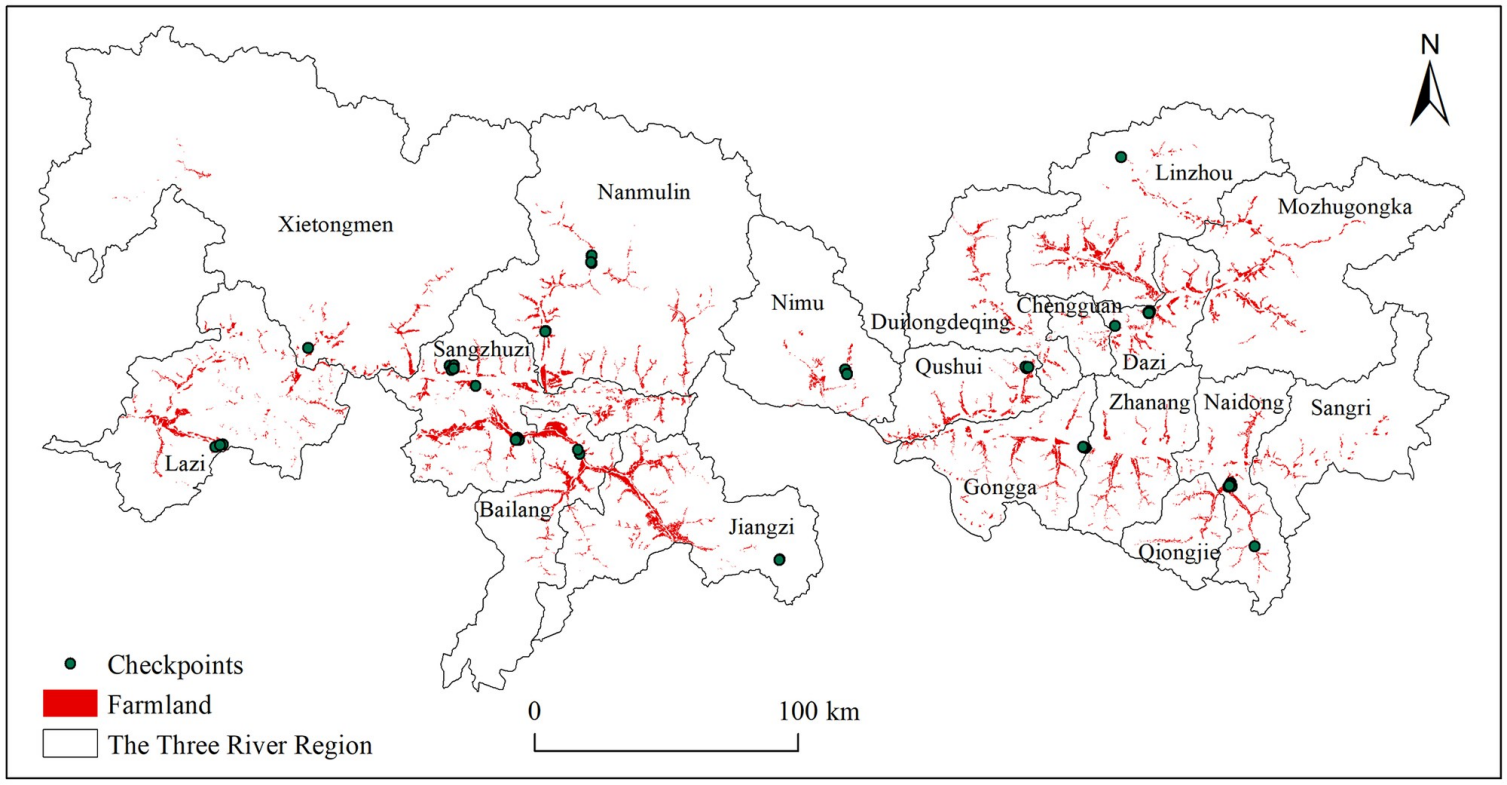
Spatial distribution of 55 farmland checkpoints in the Three River Region. The map was prepared with ArcGIS10.1 (https://resources.arcgis.com/en/help/), based on the maps of China political boundaries from the Resource and Environment Science and Data Center, CAS (https://www.resdc.cn/), with the permission of its copyright, checkpoints and farmland data from Wei et al., [34] (https://doi.pangaea.de/10.1594/PANGAEA.937400).

The 0.51–1.02 m resolution satellite images used for the visual interpretation of farmland are clearly distinguishable, ensuring the data accuracy. However, due to the lack of high-resolution images for 2000 and 2018 in some areas, the images were not in time-consistence and thus may cause some deviations, although farmland area in the TRR did not change much within a short period of 1–3 years. We randomly checked 55 plots (Fig 3) based on data collected by field surveys in 2018–2021, finding that 96.36% of the farmland parcels in 2018 were correctly identified, implying that the dataset of farmland generally has a good quality, well revealing the farmland distribution in the TRR.

The interpreted farmland area in the TRR was 219.29 k ha in 2000 and 199.89 k ha in 2018, much lower than those (254.39 k ha and 251.36 k ha) extracted from the 30 m resolution land use map, as released by the Resource and Environment Science and Data Center of the Chinese Academy of Sciences, respectively. By comparing the spatial distribution maps, we found that the discrepancies mainly occurred in small farmland parcels, of which the area was over-extracted by the 30 m resolution data. Compared with our data, the officially reported statistical farmland was under reported by 103.28 k ha (47.10%) in 2000 and 96.13 k ha (48.09%) in 2018. This is because the statistical farmland in Tibet is still based on history data that are often largely under-recorded [35].

### Farmland change and its driving factors

During 2000–2018, farmland area in the TRR showed a significant decrease trend, similar to the general trend in most regions of China [36–39], while it was contrary to the results of four existing researches on farmland change in Tibet that all showed an increase [17, 23–25]. The reason is attributed to that the data used in these researches have a coarser resolution as collected from yearbooks and 30 m resolution remotely sensed land use data, and cannot accurately reflect the actual situation. Our results indicate that the decrease of farmland in the TRR was more significant in urban areas and their surrounding counties, in accordance with previous research results [37, 38].

Farmland changes are jointly affected by natural, economic, social, and policy factors [36–38, 40]. In general, natural factors exhibiting little change in a short period and policy factors are difficult to quantify, so, this study mainly analyzed the influences of socioeconomic factors on the farmland changes. The results revealed that the economic factors including local fiscal expenditure, total investment in fixed assets, GDP, and rural disposable income per capita, were the main driving forces, together explaining 56.96% of the farmland change in the TRR. The main reason is that these factors can promote an increase in demand for non-agricultural construction land [36–38]. From 2000 to 2018, the GDP, local fiscal expenditure and total fixed asset investment increased by 26.46 times, 54.99 times and 137.26 times in the TRR, respectively. These greatly stimulated the construction of infrastructure and urban development/land expansion. Statistical data indicate that during 2005–2017, 468 km roads were constructed in Tibet, and the urban area of Lhasa and Shannan cities was expanded by 106.29% and 131.92%, respectively [41]. By rough interpretation of the high-resolution imagery data, we estimated that more than 40% of the reduced farmland was converted to built-up land during 2000–2018, and the remaining 60% was occupied by urban greenbelts and ecological restoration as triggered by the "Grain for Green Program" implemented from 2000 [42, 43]. In addition, during past 20 years, rural disposable income per capita in the TRR was greatly increased by 7.05 times, and thus raised the requirement for housing. In 2018–2020, we did three times of household interviews in Tibet, and found that most rural houses have been newly built during recent two decades, partly supported by the central government or fully aided by the brother provincial governments. Meanwhile, during 2000–2018, the GDP proportion of agriculture decreased from 53.32% to 6.32%, while that of industry and service sector increased from 13.97% to 38.64% and from 32.71% to 55.04% in the TRR, respectively. This adjustment of the economic structure increased the need of land space for non-agricultural activities and infrastructure construction, and also created more non-agricultural employment opportunities, and thus improved income and further stimulated the housing demand.

Population plays a two-way regulatory role in the farmland change. On the one hand, population growth requires more farmland to provide grain to meet the food need; and on the other hand, it increases demand for housing and infrastructure construction land [44]. During the study period, the total population increased from 825,200 in 2000 to 1,073,600 in 2018, increased by 30.10% in the TRR, which inevitably increased housing demand and thus caused farmland occupation. In addition, the urbanization level increased from 20.10% to 30.16%, increased by 10.06% in the TRR from 2000 to 2018. Associated with this change, the built-up land occupation of farmland increased.

Agricultural mechanization was also identified as a significant driving factor of the farmland change, consistent with previous research results [40]. The reason could be that the improvement of agricultural mechanization level reduced labor requirement, and thus more farmers left to seek higher income jobs in cities, resulting in some farmers converted their farmland to grassland for animal grazing.

### Farmland change impacts and implications

Farmland loss in the TRR largely occurred in urban areas and their surrounding counties and thus could have a more significant impact on grain production, as the lost farmland generally had a higher quality. Our analysis result confirmed this deduction, as the farmland reduction was totaled 8.85% between 2000 and 2018, but caused a reduction of 9.38% in the total grain production. Benefited from grain yield improvement, total grain production in the TRR still increased by 1.90% (10,219 tons) in 2018 compared to 2000. Even so, the impact of farmland reduction should not be overlooked. With the Chinese government support, the quick socioeconomic development in Tibet during recent 20 years is expected to be maintained for the next two decades, and would inevitably cause further urban encroachment to the farmland. Considering the key role of the TRR in meeting the grain demand of Tibet, it should enhance the farmland protection by stricter supervision on the farmland conversion to built-up land or to urban greenbelt, as to reduce the further reduction of fertile farmland in the areas surrounding cities. The national policy of ecological conversion of farmland that has been practiced for nearly 20 years, may need to be adjusted considering the local condition, to avoid inducing over-conversion of good quality farmland to trees, because of the financial support. In addition, more efforts and investments or subsidy are needed to improve the irrigation system, farmland quality and land management, and thus the crop productivity.

## Conclusions

From 2000 to 2018, farmland area in the TRR decreased by 8.85% (19.40 k ha), reduced by an average of 0.51% per year. A total of 50.17 k ha farmland was lost, largely occurred in urban areas and their surrounding counties due to the urban land expansion, and 30.77 k ha was newly reclaimed for crop cultivation in the rural areas of main grain producing counties. The economic development was the most important driving force of farmland change, and the agricultural progress, urbanization, and population growth played next important roles. The farmland loss had a significant impact on the grain production, causing the total production reduced by 9.38% in the TRR. Therefore, farmland protection and agricultural investment should be enhanced to control the farmland losses and to improve the productivity. This study produced highly accurate farmland data and filled the data gap in the TRR, and further detected the change during recent two decades, indicating a rather serious situation of farmland loss, although the analysis was based on data in two years without considering the dynamic process.

## Supporting information

S1 File The data used in this manuscript is provided in S1 File. (RAR)
